# The genome sequence of an ichneumonid wasp, *Tromatobia lineatoria *(Villers, 1789)

**DOI:** 10.12688/wellcomeopenres.19830.1

**Published:** 2023-08-11

**Authors:** Benjamin W. Price, Gavin R. Broad, Natural History Museum Genome Acquisition Lab, Darwin Tree of Life Barcoding collective, Wellcome Sanger Institute Tree of Life programme, Wellcome Sanger Institute Scientific Operations: DNA Pipelines collective, Tree of Life Core Informatics collective, Darwin Tree of Life Consortium

**Affiliations:** 1 Natural History Museum, London, England, UK

**Keywords:** Tromatobia lineatoria, an ichneumonid wasp, genome sequence, chromosomal, Hymenoptera

## Abstract

We present a genome assembly from an individual female *Tromatobia lineatoria* (an ichneumonid wasp; Arthropoda; Insecta; Hymenoptera; Ichneumonidae). The genome sequence is 383.6 megabases in span. Most of the assembly is scaffolded into 21 chromosomal pseudomolecules. The mitochondrial genome has also been assembled and is 23.25 kilobases in length.

## Species taxonomy

Eukaryota; Metazoa; Eumetazoa; Bilateria; Protostomia; Ecdysozoa; Panarthropoda; Arthropoda; Mandibulata; Pancrustacea; Hexapoda; Insecta; Dicondylia; Pterygota; Neoptera; Endopterygota; Hymenoptera; Apocrita; Ichneumonoidea; Ichneumonidae; Pimplinae; Ephialtini; *Tromatobia*; *Tromatobia lineatoria* (Villers, 1789) (NCBI:txid2776060).

## Background

*Tromatobia lineatoria* is an attractively marked ichneumonid wasp (‘Darwin wasp’) of the subfamily Pimplinae, usually richly patterned with red and cream against a black background. Unlike some similar *Tromatobia* species, the conspicuous yellow orbital stripe is acutely angled near the hind ocellus. Across much of Europe and the Near East, *T. lineatoria* is the most frequently encountered *Tromatobia* species, often found around houses, as its larvae feed on spider egg sacs, including those of common garden species such as *Zygiella x-notata* (Clerck) and *Araneus diadematus* Clerck. Probably occurring throughout Britain and Ireland, *T. lineatoria* adults can be found from spring to late autumn and are at least double brooded ( Fitton *et al.*, 1988). Note that in the older literature, this species was usually named *Tromatobia oculatoria*, which was an old misidentification ( Horstmann, 2001).

Although belonging to a family of parasitoid wasps, *Tromatobia* species are essentially predators. Eggs are laid within the silken egg sacs of spiders, with known hosts mainly Araneidae but also Linyphiidae, Philodromidae and Tetragnathidae. The larvae of *T. lineatoria* are usually gregarious, with brood sizes of one to six, each larva consuming spider eggs in succession. Any spider guarding the egg sac is not attacked and if any spider eggs remain, spiderlings can emerge ( Fitton *et al.*, 1988; Nielsen, 1923). Larvae from late autumn broods will feed slowly through the winter, with adults emerging in the spring ( Fitton *et al.*, 1987).

Within the subfamily Pimplinae there is a wide range of host associations and developmental biology. Townes (1969) proposed that the remarkable polysphinctines, larvae of which develop on active spiders, arose from parasitoids of silk-cocooned moths, via an intermediate life history of attacking silken egg sacs. This evolutionary pathway has been supported by phylogenetic studies ( Gauld *et al.*, 2002; Matsumoto, 2016; Spasojevic *et al.*, 2021), and *Tromatobia* occupies an intriguing phylogenetic position either as part of a radiation of spider egg sac predators ( Gauld *et al.*, 2002; Matsumoto, 2016) or as the closest relatives of *Gregopimpla*, which are gregarious parasitoids of cocooned moth pupae. Either way, the genome of *T. lineatoria*, together with genomes of polysphinctines and other related pimplines (e.g., Broad *et al.*, 2023), should help us understand some of the adaptations which enabled these transitions.

## Genome sequence report

**Figure 1.**
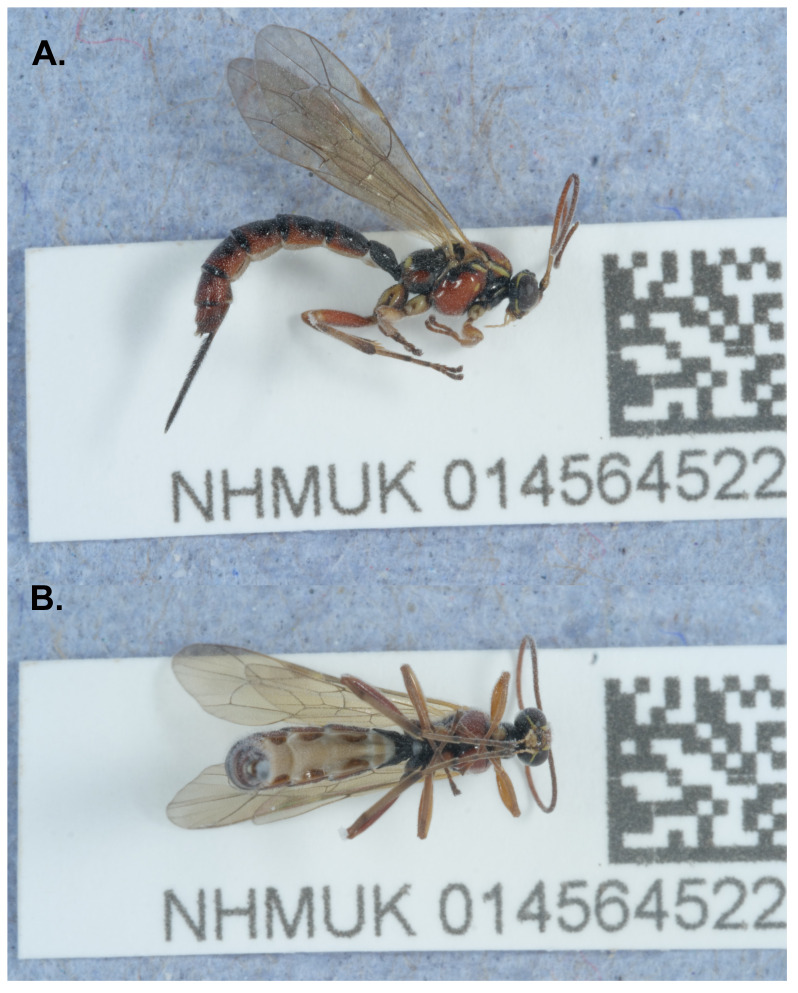
Photograph of the *Tromatobia lineatoria* (iyTroLine1) specimen used for genome sequencing. **A**. Lateral view, **B**. Ventral view.

The genome was sequenced from one female *Tromatobia lineatoria* ( Figure 1) collected from Pulborough, UK (50.96, –0.51). A total of 58-fold coverage in Pacific Biosciences single-molecule HiFi long reads was generated. Primary assembly contigs were scaffolded with chromosome conformation Hi-C data. Manual assembly curation corrected 45 missing joins or misjoins and removed one haplotypic duplication, reducing the scaffold number 16.25%, and increasing the scaffold N50 by 7.59%.

**Figure 2.**
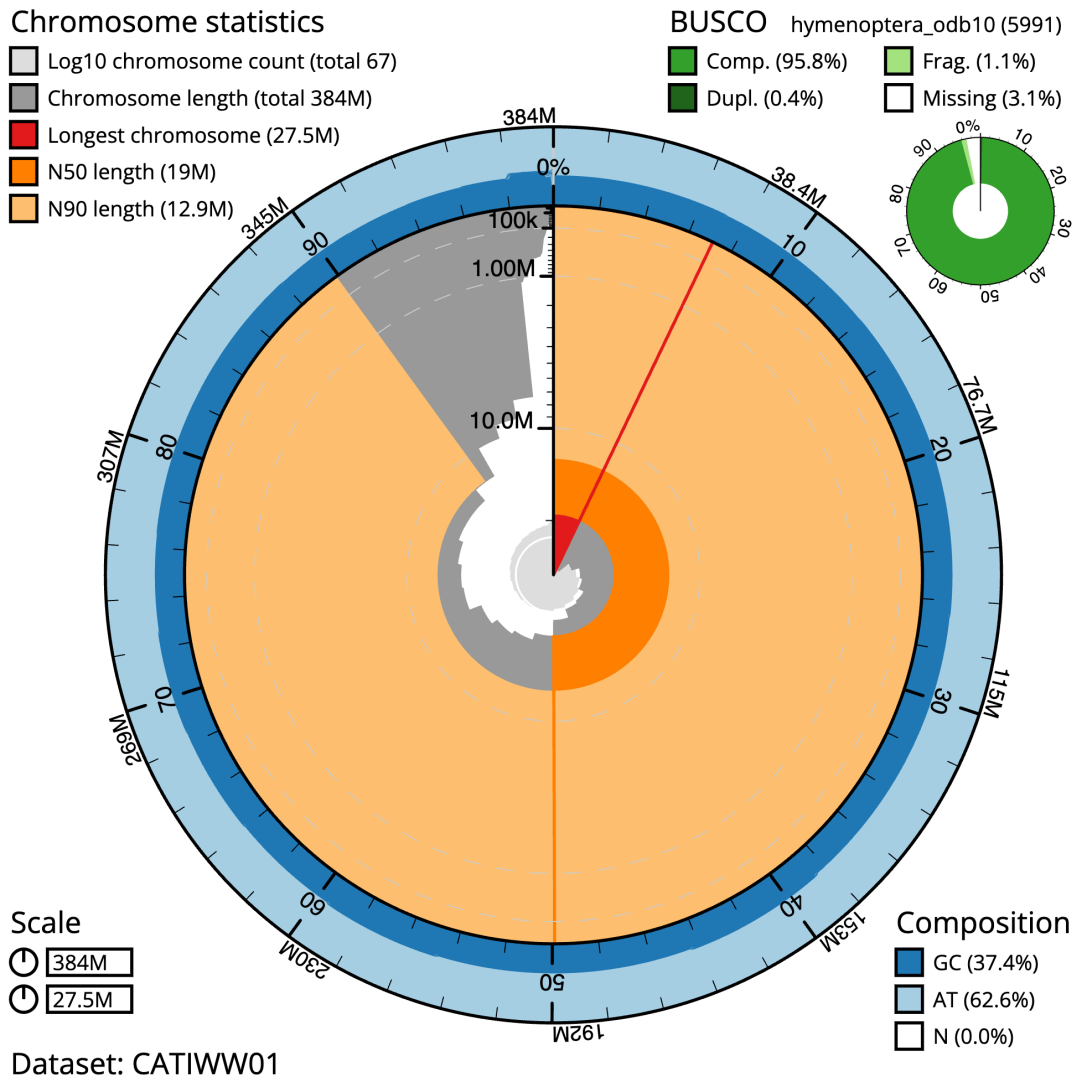
Genome assembly of *Tromatobia lineatoria*, iyTroLine1.1: metrics. The BlobToolKit Snailplot shows N50 metrics and BUSCO gene completeness. The main plot is divided into 1,000 size-ordered bins around the circumference with each bin representing 0.1% of the 383,602,644 bp assembly. The distribution of scaffold lengths is shown in dark grey with the plot radius scaled to the longest scaffold present in the assembly (27,496,960 bp, shown in red). Orange and pale-orange arcs show the N50 and N90 scaffold lengths (19,039,001 and 12,934,278 bp), respectively. The pale grey spiral shows the cumulative scaffold count on a log scale with white scale lines showing successive orders of magnitude. The blue and pale-blue area around the outside of the plot shows the distribution of GC, AT and N percentages in the same bins as the inner plot. A summary of complete, fragmented, duplicated and missing BUSCO genes in the hymenoptera_odb10 set is shown in the top right. An interactive version of this figure is available at https://blobtoolkit.genomehubs.org/view/iyTroLine1.1/dataset/CATIWW01/snail.

The final assembly has a total length of 383.6 Mb in 66 sequence scaffolds with a scaffold N50 of 19.0 Mb ( Table 1). Most (98.1%) of the assembly sequence was assigned to 21 chromosomal-level scaffolds. Chromosome-scale scaffolds confirmed by the Hi-C data are named in order of size ( Figure 2– Figure 5; Table 2). The order and orientation of contigs on SUPER_7 between 17Mb and 21 Mb is uncertain. While not fully phased, the assembly deposited is of one haplotype. Contigs corresponding to the second haplotype have also been deposited. The mitochondrial genome was also assembled and can be found as a contig within the multifasta file of the genome submission.

**Table 1.** Genome data for *Tromatobia lineatoria*, iyTroLine1.1.

Project accession data		
Assembly identifier	iyTroLine1.1	
Species	*Tromatobia lineatoria*	
Specimen	iyTroLine1	
NCBI taxonomy ID	2776060	
BioProject	PRJEB59790	
BioSample ID	SAMEA111458168	
Isolate information	iyTroLine1, female: head and thorax (DNA sequencing and Hi-C scaffolding)	
Assembly metrics *		*Benchmark*
Consensus quality (QV)	62.4	*≥ 50*
*k*-mer completeness	100%	*≥ 95%*
BUSCO **	C:95.8%[S:95.4%,D:0.4%], F:1.1%,M:3.1%,n:5,991	*C ≥ 95%*
Percentage of assembly mapped to chromosomes	98.1%	*≥ 95%*
Sex chromosomes	-	*localised homologous * *pairs*
Organelles	Mitochondrial genome assembled	*complete single alleles*
Raw data accessions		
PacificBiosciences SEQUEL II	ERR10879932	
Hi-C Illumina	ERR10890735	
Genome assembly		
Assembly accession	GCA_949699805.1	
*Accession of alternate * *haplotype*	GCA_949699785.1	
Span (Mb)	383.6	
Number of contigs	218	
Contig N50 length (Mb)	3.8	
Number of scaffolds	66	
Scaffold N50 length (Mb)	19.0	
Longest scaffold (Mb)	27.5	

* Assembly metric benchmarks are adapted from column VGP-2020 of “Table 1: Proposed standards and metrics for defining genome assembly quality” from ( Rhie *et al.*, 2021). ** BUSCO scores based on the hymenoptera_odb10 BUSCO set using v5.3.2. C = complete [S = single copy, D = duplicated], F = fragmented, M = missing, n = number of orthologues in comparison. A full set of BUSCO scores is available at https://blobtoolkit.genomehubs.org/view/iyTroLine1.1/dataset/CATIWW01/busco.

**Figure 3.**
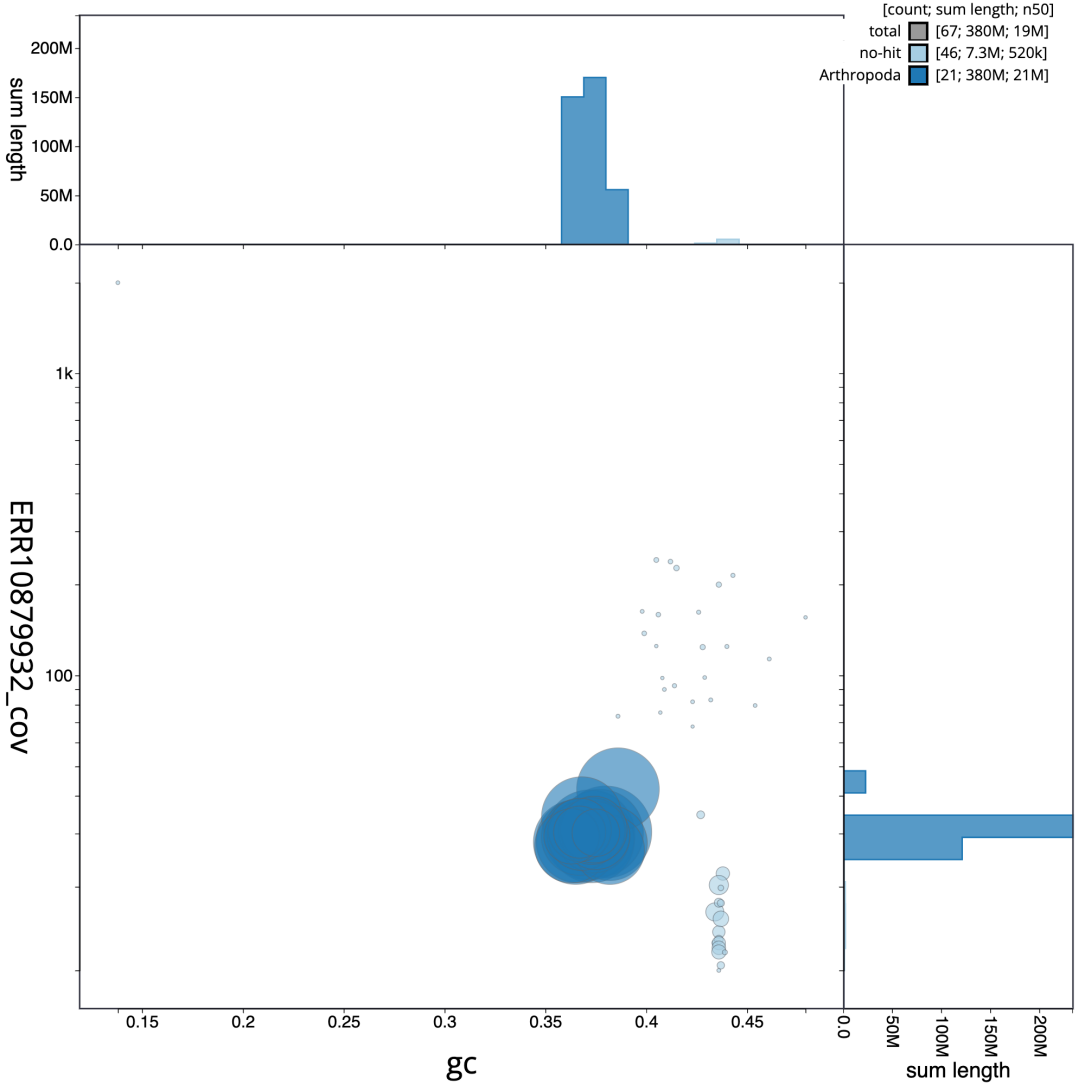
Genome assembly of *Tromatobia lineatoria*, iyTroLine1.1: BlobToolKit GC-coverage plot. Scaffolds are coloured by phylum. Circles are sized in proportion to scaffold length. Histograms show the distribution of scaffold length sum along each axis. An interactive version of this figure is available at https://blobtoolkit.genomehubs.org/view/iyTroLine1.1/dataset/CATIWW01/blob.

**Figure 4.**
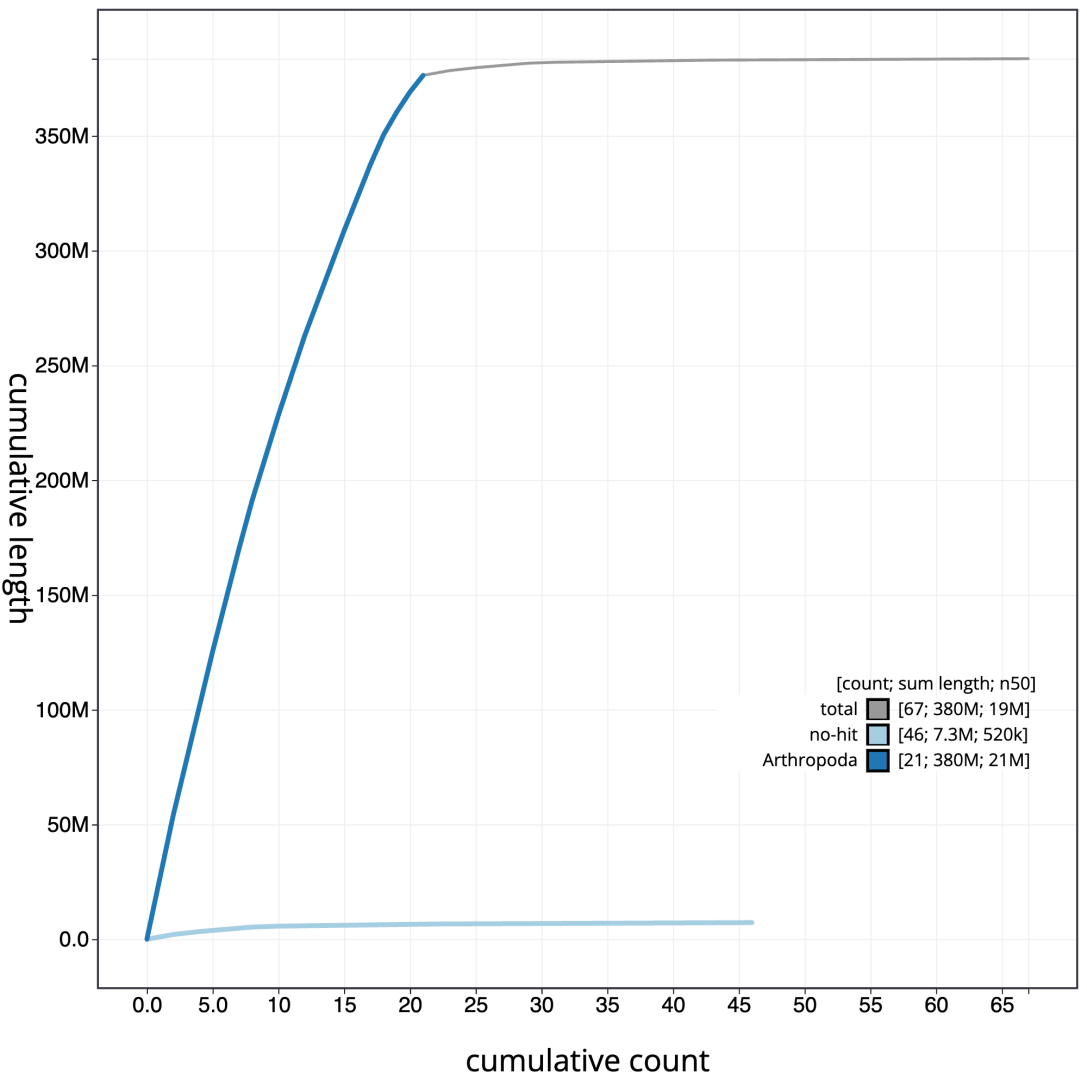
Genome assembly of *Tromatobia lineatoria*, iyTroLine1.1: BlobToolKit cumulative sequence plot. The grey line shows cumulative length for all scaffolds. Coloured lines show cumulative lengths of scaffolds assigned to each phylum using the buscogenes taxrule. An interactive version of this figure is available at https://blobtoolkit.genomehubs.org/view/iyTroLine1.1/dataset/CATIWW01/cumulative.

**Figure 5.**
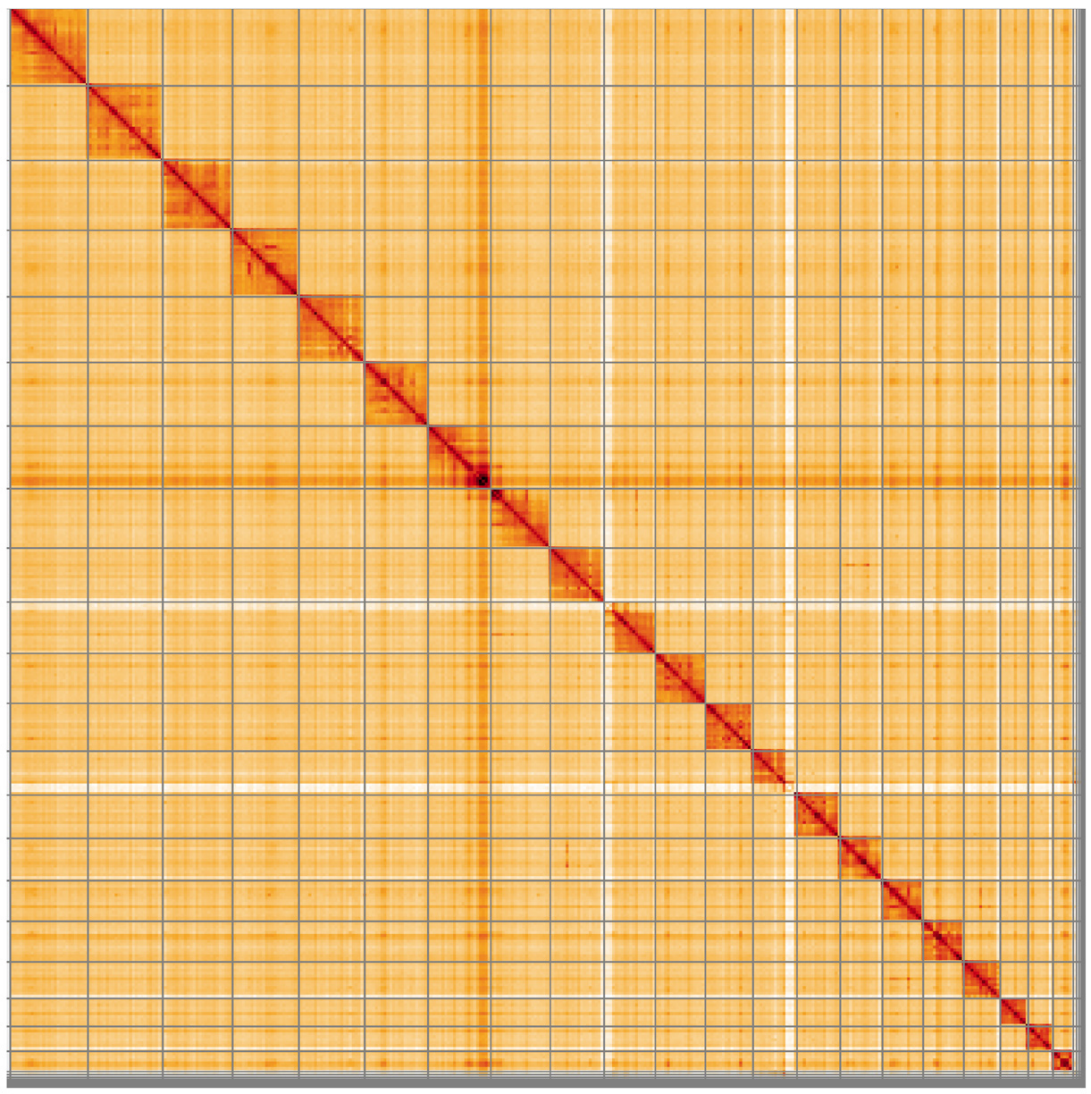
Genome assembly of *Tromatobia lineatoria*, iyTroLine1.1: Hi-C contact map of the iyTroLine1.1 assembly, visualised using HiGlass. Chromosomes are shown in order of size from left to right and top to bottom. An interactive version of this figure may be viewed at https://genome-note-higlass.tol.sanger.ac.uk/l/?d=cZepPc8bQ0qO5ZccVO9iEQ.

**Table 2.** Chromosomal pseudomolecules in the genome assembly of *Tromatobia lineatoria*, iyTroLine1.

INSDC accession	Chromosome	Length (Mb)	GC%
OX453034.1	1	27.5	38.0
OX453035.1	2	26.45	37.0
OX453036.1	3	24.67	37.5
OX453037.1	4	23.55	37.5
OX453038.1	5	23.24	36.5
OX453039.1	6	22.47	36.5
OX453040.1	7	22.21	38.5
OX453041.1	8	21.05	37.0
OX453042.1	9	19.04	36.5
OX453043.1	10	18.17	38.0
OX453044.1	11	17.7	36.5
OX453045.1	12	16.94	37.5
OX453046.1	13	15.52	38.0
OX453047.1	14	15.35	37.0
OX453048.1	15	14.94	37.5
OX453049.1	16	14.41	37.5
OX453050.1	17	14.4	37.5
OX453051.1	18	12.93	36.5
OX453052.1	19	9.89	36.5
OX453053.1	20	8.71	36.5
OX453054.1	21	7.21	37.5
OX453055.1	MT	0.02	14.0

The estimated Quality Value (QV) of the final assembly is 62.4 with *k*-mer completeness of 100%, and the assembly has a BUSCO v5.3.2 completeness of 95.8% (single = 95.4%, duplicated = 0.4%), using the hymenoptera_odb10 reference set ( *n* = 5,991).

Metadata for specimens, spectral estimates, sequencing runs, contaminants and pre-curation assembly statistics can be found at https://links.tol.sanger.ac.uk/species/2776060.

## Methods

### Sample acquisition and nucleic acid extraction

A female *Tromatobia lineatoria* (specimen ID NHMUK014564522, individual iyTroLine1) was collected by hand from Pulborough, UK (latitude 50.96, longitude –0.51) on 2021-10-30 by Benjamin Price (Natural History Museum). The specimen was identified by Gavin Broad (Natural History Museum) and preserved by dry freezing at –80°C.

DNA was extracted at the Tree of Life laboratory, Wellcome Sanger Institute (WSI). The iyTroLine1 sample was weighed and dissected on dry ice with tissue set aside for Hi-C sequencing. Head and thorax tissue was disrupted using a Nippi Powermasher fitted with a BioMasher pestle. High molecular weight (HMW) DNA was extracted using the Qiagen MagAttract HMW DNA extraction kit. HMW DNA was sheared into an average fragment size of 12–20 kb in a Megaruptor 3 system with speed setting 30. Sheared DNA was purified by solid-phase reversible immobilisation using AMPure PB beads with a 1.8X ratio of beads to sample to remove the shorter fragments and concentrate the DNA sample. The concentration of the sheared and purified DNA was assessed using a Nanodrop spectrophotometer and Qubit Fluorometer and Qubit dsDNA High Sensitivity Assay kit. Fragment size distribution was evaluated by running the sample on the FemtoPulse system.

### Sequencing

Pacific Biosciences HiFi circular consensus DNA sequencing libraries were constructed according to the manufacturers’ instructions. DNA sequencing was performed by the Scientific Operations core at the WSI on a Pacific Biosciences SEQUEL II (HiFi) instrument. Hi-C data were also generated from remaining head/thorax tissue of iyTroLine1 using the Arima2 kit and sequenced on the Illumina NovaSeq 6000 instrument.

### Genome assembly, curation and evaluation

Assembly was carried out with Hifiasm ( Cheng *et al.*, 2021) and haplotypic duplication was identified and removed with purge_dups ( Guan *et al.*, 2020). The assembly was then scaffolded with Hi-C data ( Rao *et al.*, 2014) using YaHS ( Zhou *et al.*, 2023). The assembly was checked for contamination and corrected as described previously ( Howe *et al.*, 2021). Manual curation was performed using HiGlass ( Kerpedjiev *et al.*, 2018) and Pretext ( Harry, 2022). The mitochondrial genome was assembled using MitoHiFi ( Uliano-Silva *et al.*, 2023), which runs MitoFinder ( Allio *et al.*, 2020) or MITOS ( Bernt *et al.*, 2013) and uses these annotations to select the final mitochondrial contig and to ensure the general quality of the sequence.

A Hi-C map for the final assembly was produced using bwa-mem2 ( Vasimuddin *et al.*, 2019) in the Cooler file format ( Abdennur & Mirny, 2020). To assess the assembly metrics, the *k*-mer completeness and QV consensus quality values were calculated in Merqury ( Rhie *et al.*, 2020). This work was done using Nextflow ( Di Tommaso *et al.*, 2017) DSL2 pipelines “sanger-tol/readmapping” ( Surana *et al.*, 2023a) and “sanger-tol/genomenote” ( Surana *et al.*, 2023b). The genome was analysed within the BlobToolKit environment ( Challis *et al.*, 2020) and BUSCO scores ( Manni *et al.*, 2021; Simão *et al.*, 2015) were calculated.

Table 3 contains a list of relevant software tool versions and sources.

**Table 3.** Software tools: versions and sources.

Software tool	Version	Source
BlobToolKit	4.1.7	https://github.com/blobtoolkit/blobtoolkit
BUSCO	5.3.2	https://gitlab.com/ezlab/busco
Hifiasm	0.16.1-r375	https://github.com/chhylp123/hifiasm
HiGlass	1.11.6	https://github.com/higlass/higlass
Merqury	MerquryFK	https://github.com/thegenemyers/MERQURY.FK
MitoHiFi	2	https://github.com/marcelauliano/MitoHiFi
PretextView	0.2	https://github.com/wtsi-hpag/PretextView
purge_dups	1.2.3	https://github.com/dfguan/purge_dups
sanger-tol/genomenote	v1.0	https://github.com/sanger-tol/genomenote
sanger-tol/readmapping	1.1.0	https://github.com/sanger-tol/readmapping/tree/1.1.0
YaHS	1.2a	https://github.com/c-zhou/yahs

### Wellcome Sanger Institute – Legal and Governance

The materials that have contributed to this genome note have been supplied by a Darwin Tree of Life Partner. The submission of materials by a Darwin Tree of Life Partner is subject to the **‘Darwin Tree of Life Project Sampling Code of Practice’**, which can be found in full on the Darwin Tree of Life website here. By agreeing with and signing up to the Sampling Code of Practice, the Darwin Tree of Life Partner agrees they will meet the legal and ethical requirements and standards set out within this document in respect of all samples acquired for, and supplied to, the Darwin Tree of Life Project. 

Further, the Wellcome Sanger Institute employs a process whereby due diligence is carried out proportionate to the nature of the materials themselves, and the circumstances under which they have been/are to be collected and provided for use. The purpose of this is to address and mitigate any potential legal and/or ethical implications of receipt and use of the materials as part of the research project, and to ensure that in doing so we align with best practice wherever possible. The overarching areas of consideration are:

•   Ethical review of provenance and sourcing of the material

•   Legality of collection, transfer and use (national and international) 

Each transfer of samples is further undertaken according to a Research Collaboration Agreement or Material Transfer Agreement entered into by the Darwin Tree of Life Partner, Genome Research Limited (operating as the Wellcome Sanger Institute), and in some circumstances other Darwin Tree of Life collaborators.

## Data Availability

European Nucleotide Archive: *Tromatobia lineatoria*. Accession number PRJEB59790; https://identifiers.org/ena.embl/PRJEB59790. ( Wellcome Sanger Institute, 2023). The genome sequence is released openly for reuse. The *Tromatobia lineatoria* genome sequencing initiative is part of the Darwin Tree of Life (DToL) project. All raw sequence data and the assembly have been deposited in INSDC databases. The genome will be annotated using available RNA-Seq data and presented through the Ensembl pipeline at the European Bioinformatics Institute. Raw data and assembly accession identifiers are reported in Table 1.

## Author information

Members of the Natural History Museum Genome Acquisition Lab are listed here: https://doi.org/10.5281/zenodo.4790042.

Members of the Darwin Tree of Life Barcoding collective are listed here: https://doi.org/10.5281/zenodo.4893703.

Members of the Wellcome Sanger Institute Tree of Life programme are listed here: https://doi.org/10.5281/zenodo.4783585.

Members of Wellcome Sanger Institute Scientific Operations: DNA Pipelines collective are listed here: https://doi.org/10.5281/zenodo.4790455.

Members of the Tree of Life Core Informatics collective are listed here: https://doi.org/10.5281/zenodo.5013541.

Members of the Darwin Tree of Life Consortium are listed here: https://doi.org/10.5281/zenodo.4783558.
