# Supplementary material for: The genome sequence of an ichneumonid wasp, Tromatobia lineatoria (Villers, 1789)
Source: Wellcome Open Res. 2023 Aug 11;8:339. [Version 1] doi: 10.12688/wellcomeopenres.19830.1 (PMC11282395; doi:10.12688/wellcomeopenres.19830.1)
Supplement: Peer review history

# Reviewer report: Reviewer response for version 1

**DOI:** 10.21956/wellcomeopenres.21962.r87624

**Published:** 2024-07-26

**Authors:** Mabel Alvarado

**Affiliations:** 1 Universidad Nacional Mayor de San Marcos, Lima District, Lima Region, Peru

Greetings,

 The manuscript provides a much-needed information, and it will provide the base for future research on Ichneumonidae wasps. In general, it has a similar structure to previous articles in the topic which make it easier to compare. Additionally, it is clearly written and makes it easy to follow and understand.

Are sufficient details of methods and materials provided to allow replication by others?

Yes

Is the rationale for creating the dataset(s) clearly described?

Yes

Are the datasets clearly presented in a useable and accessible format?

Yes

Are the protocols appropriate and is the work technically sound?

Yes

Reviewer Expertise:

Systematics, evolution

I confirm that I have read this submission and believe that I have an appropriate level of expertise to confirm that it is of an acceptable scientific standard.

# Reviewer report: Reviewer response for version 1

**DOI:** 10.21956/wellcomeopenres.21962.r88838

**Published:** 2024-07-26

**Authors:** Zoltán Vas

**Affiliations:** 1 Hungarian Natural History Museum, Budapest, Hungary

I am glad to see that more and more genome sequence of ichneumon wasps species are published. These, including the present study, are important footsteps to untangle and understand the evolutionary history of this hyperdiverse family. I agree with the arguments of the authors why the phylogenetic position of Tromatobia lineatoria is important, so why this species is indeed a good candidate for genome sequencing. 

 Being a morphology-based, traditional taxonomist, I am not completely up to date about the used molecular methodology, so it is partly beyond my abilities to evaluate. Though, the same lab has published sevaral similar, highly regarded papers, so I feel safe to say that everything is fine here.

Are sufficient details of methods and materials provided to allow replication by others?

Yes

Is the rationale for creating the dataset(s) clearly described?

Yes

Are the datasets clearly presented in a useable and accessible format?

Yes

Are the protocols appropriate and is the work technically sound?

Yes

Reviewer Expertise:

traditional, morphology-based taxonomy of Ichneumonidae

I confirm that I have read this submission and believe that I have an appropriate level of expertise to confirm that it is of an acceptable scientific standard.
